# Exploratory assessment of an endolaryngeal surgical model with a novel hyperangulated 3D printed laryngoscope

**DOI:** 10.1007/s00405-025-09514-6

**Published:** 2025-06-14

**Authors:** Viola D. Hahn, Gabriel L. Gschwend, Linus L. Kienle, Leon R. Schild, Jens Greve, Thomas K. Hoffmann, Patrick J. Schuler, Felix Böhm

**Affiliations:** 1 Department of Otorhinolaryngology, Head and Neck Surgery, University Medical Centre Ulm, Frauensteige 12, 89075 Ulm, Germany; 2 Department of Otorhinolaryngology, University Medical Centre Heidelberg, Im Neuenheimer Feld 400, 69120 Heidelberg, Germany

**Keywords:** Early laryngeal cancer, Head and neck surgery, Transoral surgery, Video laryngoscope, Surgical training, Porcine model

## Abstract

**Objective:**

This study introduces a hybrid training model for endolaryngeal surgeries integrating a porcine larynx into a plastic airway manikin. The study explores interventions with a conventional rigid laryngoscope as well as with the novel hyperangulated laryngoscope for surgery (sMAC).

**Methods:**

We integrated a porcine larynx into a plastic airway manikin with blue markings on vocal folds simulating lesions and utilized a conventional rigid laryngoscope and the sMAC for endolaryngeal cordectomies and biopsies.

**Results:**

The setup provided clear visual exposure of the glottic plane, enabling precise surgical interventions. Trained surgeons and residents successfully performed cordectomies and biopsies on the model.

**Conclusion:**

The hybrid model offers a realistic, cost-effective solution for training and testing endolaryngeal surgical procedures, particularly with the sMAC. Despite anatomical differences between porcine and human larynges, the model’s affordability and tissue similarity render it a valuable training tool. Further studies are warranted to evaluate its efficacy for training.

## Introduction

For early-stage laryngeal cancer, transoral laser microsurgery (TLM) or radiation therapy are established therapies [1, 2]. TLM is typically performed using a rigid operating laryngoscope, which requires not only adequate mouth opening and sufficient cervical spine mobility, but also favorable anatomical conditions that allow for a direct line of sight to the glottic plane. In approximately 10–20% of patients surgeons encounter challenges in accessing their airways, stemming from factors like limited cervical spine mobility, post-radiation changes, macroglossia, or trismus [3–5]. In such cases, performing TLM with a straight tube is difficult or impossible. To date there are some innovations that try to address this problem. While Transoral Robotic Surgery (TORS) has been successfully introduced for treating head and neck cancers in the oropharynx and supraglottic larynx, it has proven challenging to adapt for surgeries in the glottic plane [6]. A notable attempt to address this challenge was the Flex^®^ robotic system from Medrobotics, designed for robotic endoscopic procedures in the larynx [7]. But in this system and others similar products, the robotic handling is time-consuming and the operating costs are high. To address this problem, a novel instrument in form of a hyperangulated laryngoscope for surgery -the sMAC - was developed by our working group, specifically designed to facilitate transoral microsurgery in patients with anatomically difficult airways. The development of the sMAC drew on several concepts of the Flex^®^ robotic system, including the strategic arrangement of instruments and a curved design that smoothly adapts to the anatomy of the tongue and larynx, while aiming to reduce complexity and enhance cost efficiency. This innovation is based on a video laryngoscope, initially designed for managing difficult intubations, with the inclusion of additional working channels for surgical tools. It can be 3D printed and is therefore cost effective [8–11]. 

In contrast to conventional rigid laryngoscopes, which rely on a linear trajectory and are often unsuitable for patients with anatomical restrictions, the sMAC system employs an anatomically adaptive curvature that permits indirect yet effective visualization and access to the glottic plane. Due to the use of 3D printing in the production of the sMAC, its shape can be adapted to the patient-specific anatomy during the printing process. This design enables transoral microsurgery in cases previously deemed inoperable via traditional approaches. Compared to robotic systems, the sMAC offers significantly lower costs, faster setup, and direct manual control, preserving haptic feedback and surgical familiarity. Its handheld form factor allows seamless integration into existing workflows, and its modularity supports wide applicability across diverse clinical and educational environments. These advantages position the sMAC as an innovative and practical tool for expanding the indications and accessibility of transoral laryngeal surgery.

This study endeavors to find a suitable model for practicing transoral microsurgery with a conventional rigid laryngoscope as well as with the sMAC. Therefore, a model with a realistic airway anatomy is needed that allows to perform surgical interventions at the vocal folds, while still being easily available and affordable. The most realistic preclinical endolaryngeal surgery models are those of human body donors. However, their cost and limited availability pose significant challenges. Plastic manikins can be used to expose the glottic plane with a realistic human airway but fall short when it comes to testing surgical procedures or biopsies. While animal models exhibit anatomical similarities to the human larynx and are suitable for preclinical trials, differences in the upper airway anatomy and oral cavity persist. However, porcine larynges are an established choice for preclinical evaluation of laryngeal surgical procedures. Various studies have used porcine larynx in different holders for training microlaryngeal surgeries [12–14]. Some studies used manikins in combination with porcine larynx to add the perks of a realistic human airway access [15, 16]. 

To combine the advantages of a porcine larynx with the advantages of a plastic airway trainer, we integrated a porcine larynx into a plastic airway model of a human head. We hypothesized that the model allows to perform surgical interventions with the newly developed sMAC as well as the exploration of the glottic plane with the conventional rigid operating laryngoscope and therefore the possibility to use the model as a training device for residents. This is achieved by providing a lifelike airway for the precise placement of laryngoscopes, as well as presenting glottic planes comprised of fresh tissue, which meets the demands for surgical interventions.

## Materials and methods

### Porcine larynx– manikin head model

**Fig. 1:**
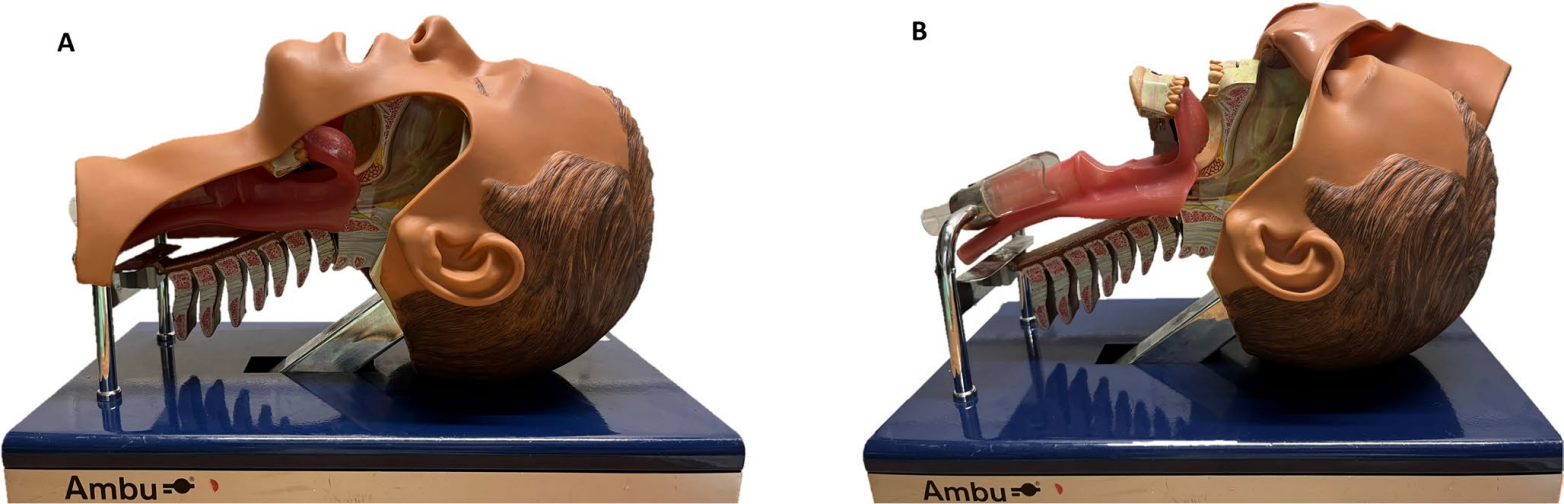
Side view of the Ambu^®^ Airway Management Trainer. **(A)** Intact Ambu^®^ Airway Management Trainer. (**B)** Plastic skin reflected to expose the larynx and pharynx

A hybrid model that integrates a porcine larynx into a plastic airway manikin was developed. As manikin a standard plastic head model for intubation training from Ambu (Ambu^®^ Airway Management Trainer) (Fig. 1) was used.

**Fig. 2:**
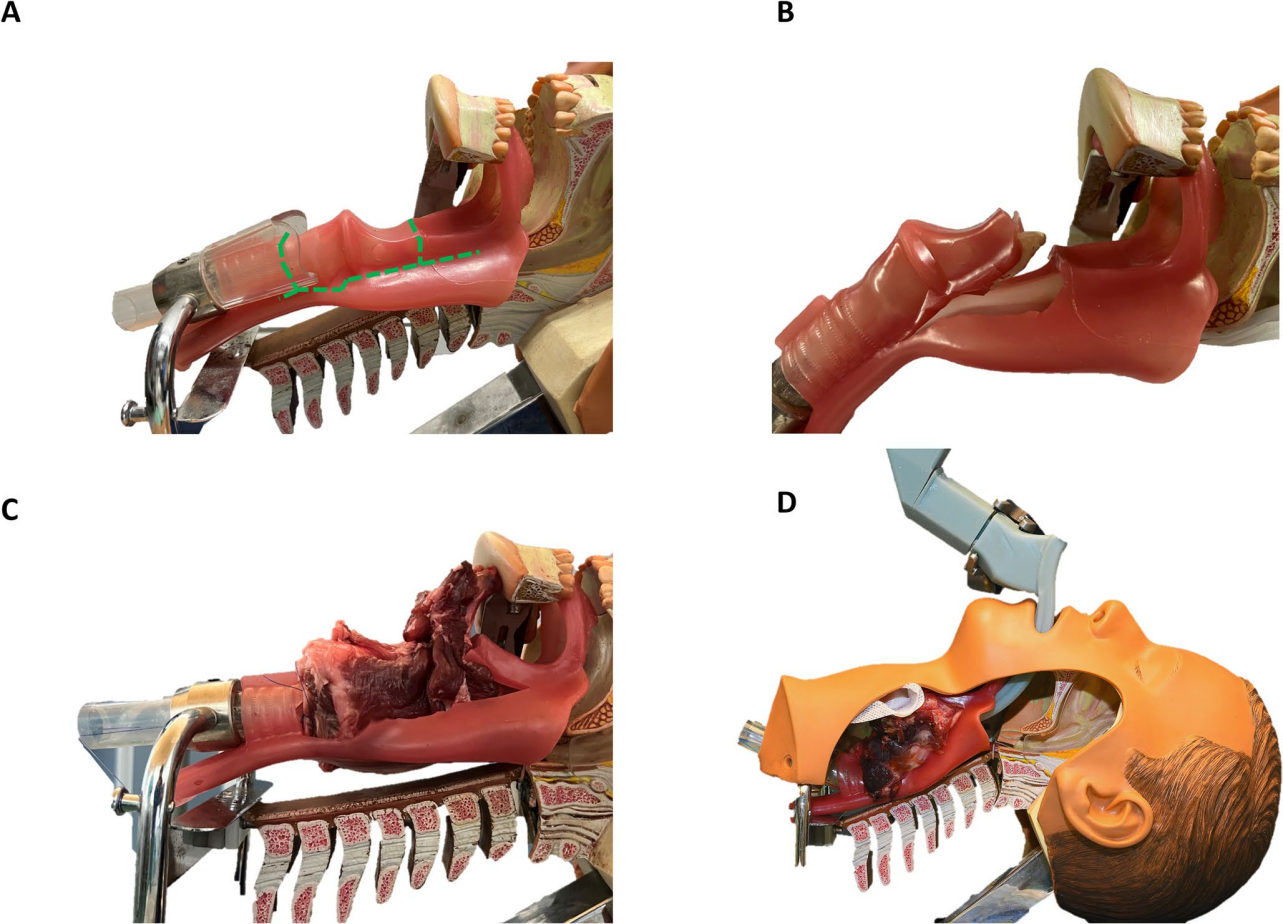
Integration of a porcine larynx into the plastic tube of the esophagus and larynx prior **(A**,** B)** and after **(C)** removal of the plastic structures of the Ambu^®^ Airway Management Trainer. The green dotted line **(A)** shows the cutting line. **(B)** Shows the rostral cut around the plastic larynx. **(C)** Incorporated porcine larynx with hyoid bone folded forward and clamped in the pharynx tube and the caudal thread loop that fixes the larynx to the metal frame. **(D)** sMAC inserted into the porcine larynx-manikin model

The hybrid model was developed by modifying the manikin head to incorporate a porcine larynx. To achieve this, certain portions of the plastic model’s larynx and select sections of the pharynx were carefully excised to create adequate room while maintaining the necessary structural integrity to support the animal tissue (Fig. 2).

As animal tissue the porcine larynges were chosen. They resemble human larynx structurally more than the larynx of other animals like sheep or dogs [17, 18]. The specimens included the larynx, the trachea, the esophagus and base of the tongue. They were stored at -22 °C and required approximately 5 h to thaw, followed by 15–30 min of preparation for integration into the model. The porcine larynges were prepared by removing the trachea, the caudal esophagus and the perilaryngeal and periesophageal tissue and muscles. The tissue around the hyoid bone was thinned by removing portions of the tongue and muscle tissue. Next, a suture was applied to the caudal cartilaginous part of the larynx, creating a thread loop with two stitches. This loop served to secure the caudal pig larynx to the metal frame through the plastic tube for the tracheal component of the head model. The porcine larynges were prepared 2 days before the experiment, frozen again and thawed the night before. Then they were installed in the head model. The upper portion of the larynx was affixed by folding the hyoid bone over and clamping the tissue within the plastic pharynx (as depicted in Fig. 2). This method effectively secured the porcine larynx and positioned its epiglottis in a forward-folded manner.

### sMAC system

**Fig. 3:**
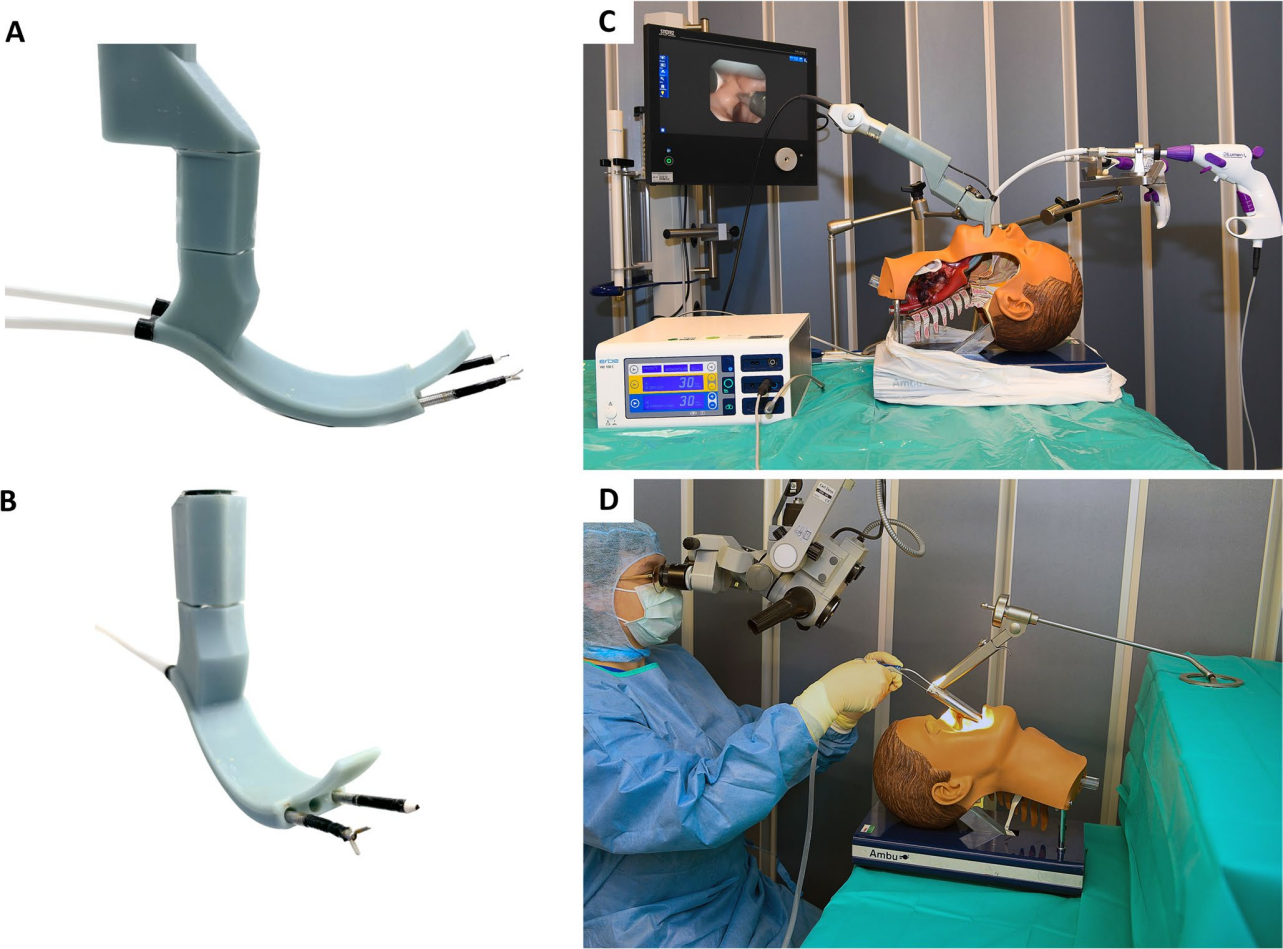
**(A**,** B)** sMAC with inserted surgical instruments. **(A)** Right-side view with the empty holder for the flexible endoscope. **(B)** Right oblique view with empty endoscope channel. **(C)** Experimental setup: Operating table with porcine larynx-manikin model. The sMAC system is placed. The glottic plane is visualized with the Storz flexible endoscope through the middle working channel, the surgical instruments namely the flexible grasper and flexible monopolar needle of Lumendi, are inserted in the outer working channels. **(D)** Conventional rigid laryngoscope inserted into a manikin model

The sMAC instrument is based on the shape of a hyperangulated videolaryngoscope (cMAC, Karl Storz), with three additional channels: Two working channels for surgical instruments and one channel for visualization with a flexible endoscope, as well as a holder for the endoscope attached to the handle (Fig. 3.A./B.). The modifications were implemented by computer-aided design (CAD) and 3D printing. The exact structure and function have already been published previously [10]. The 3D-printed sMAC was fastened with an articulated stand (28272 HA, Karl Storz) in combination with a clamping jaw (28272 UFN, Karl Storz) at an operating table. A flexible zero-degree endoscope 11101HDK (Karl Storz, Tuttlingen, Germany) was placed in the central working channel for visualization. The set-up is shown in Fig. 3C.

### Conventional laryngeal microsurgery equipment

For the conducted experiments, a conventional Kleinsasser tube (8590 A, Karl Storz Endoskope, Tuttlingen, Germany) was employed. In addition, standard instruments from Karl Storz for microscopic laryngeal surgery were utilized. These included a variety of forceps, such as curved and straight scissors, small double spoon forceps, and other specialized tools designed for precision in delicate surgical procedures. The microscope (OPMI ORL S5, Carl Zeiss, Oberkochen, Germany) of our temporal bone lab was used.

The laryngoscope was secured using a standard support device (8575 K, Karl Storz Endoskope, Tuttlingen, Germany), typically affixed either to the patient’s chest or to a dedicated plate on the operating table. This setup ensured stability and optimal positioning during the surgical interventions, facilitating precise manipulation and observation under the microscope. The setup is shown in Fig. 3D.

Additionally, appropriate single-use suction devices (Spiggle & Theis Medizintechnik, Dieburg, Germany) were employed to maintain a clear surgical field, ensuring unobstructed visibility and safety throughout the procedures. The setup is shown in Fig. 3D.

### Performed interventions

The exposition of the glottic plane was conducted with the conventional rigid operating laryngoscope (Kleinsasser) and the 3D printed hyperangulated sMAC system. In between the exchanges of porcine larynges, residents and trained head and neck surgeons carried out cordectomies on the rostral vocal folds, as well as cordectomies after marking one vocal fold with methylene blue dye. These procedures were performed using the sMAC system and the conventional operating laryngoscope (Kleinsasser).

### Ethical considerations

Informed consent was obtained from all participating surgeons, and steps were taken to anonymize any potentially identifiable information to ensure confidentiality and privacy. As this study exclusively involved surgeons as participants and did not involve other human subjects, ethical approval was not sought.

## Results

**Fig. 4:**
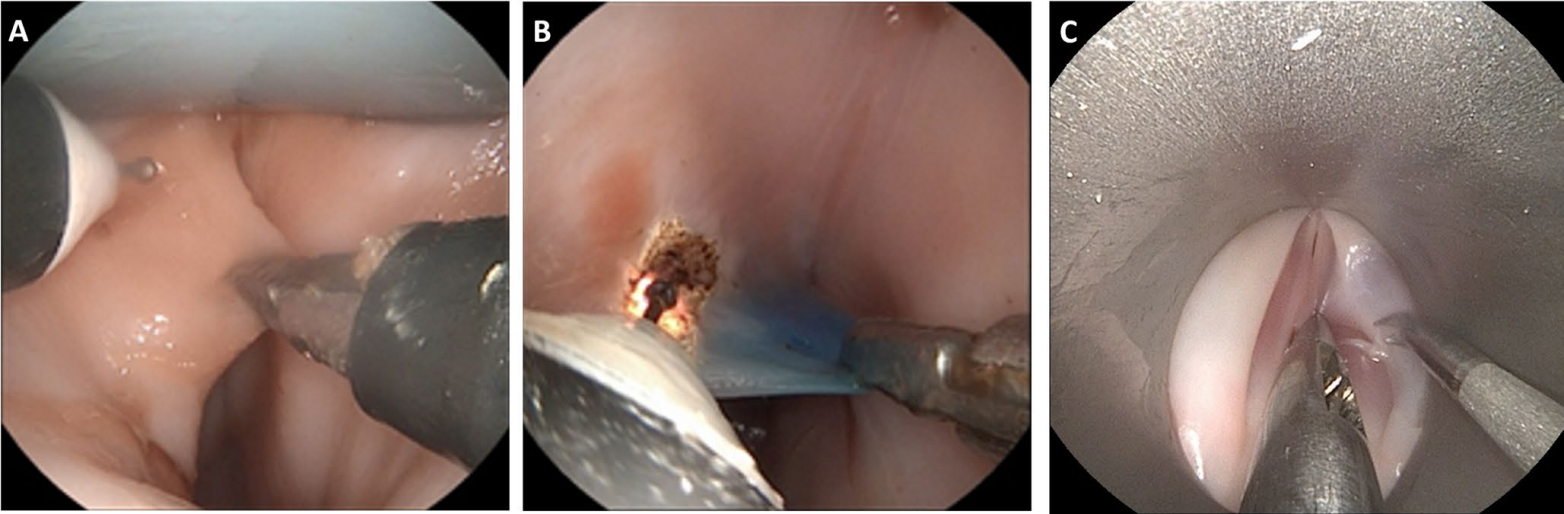
Endolaryngeal view with the sMAC in two different porcine larynxes. (**A)** Unmarked vocal folds prior to the cordectomies. **(B)** The left vocal fold was priorly marked blue with methylene blue. **(C)** Endolaryngeal view with a conventional rigid laryngoscope inserted into the porcine-larynx-model

The assembly of the entire setup went smoothly and without taking up much space. In all larynges tested, the sMAC system consistently provided clear visual exposure of the glottic plane (Fig. 4.A./B.). The visualization with the straight laryngoscope was also possible but often more difficult than with the sMAC (Fig. 4.C). The exchanges of the porcine larynx and fixation into the model head were quick and easy to carry out.

All structures of the larynx, including the epiglottis, vocal folds, vestibular folds or more correctly rostral vocal folds, the subglottic region, the anterior commissure and the postcricoid region could be identified and reached with the instruments. Inserting the endoscope and instruments, as well as guiding the instruments within the pharynx, is very intuitive.

**Fig. 5:**
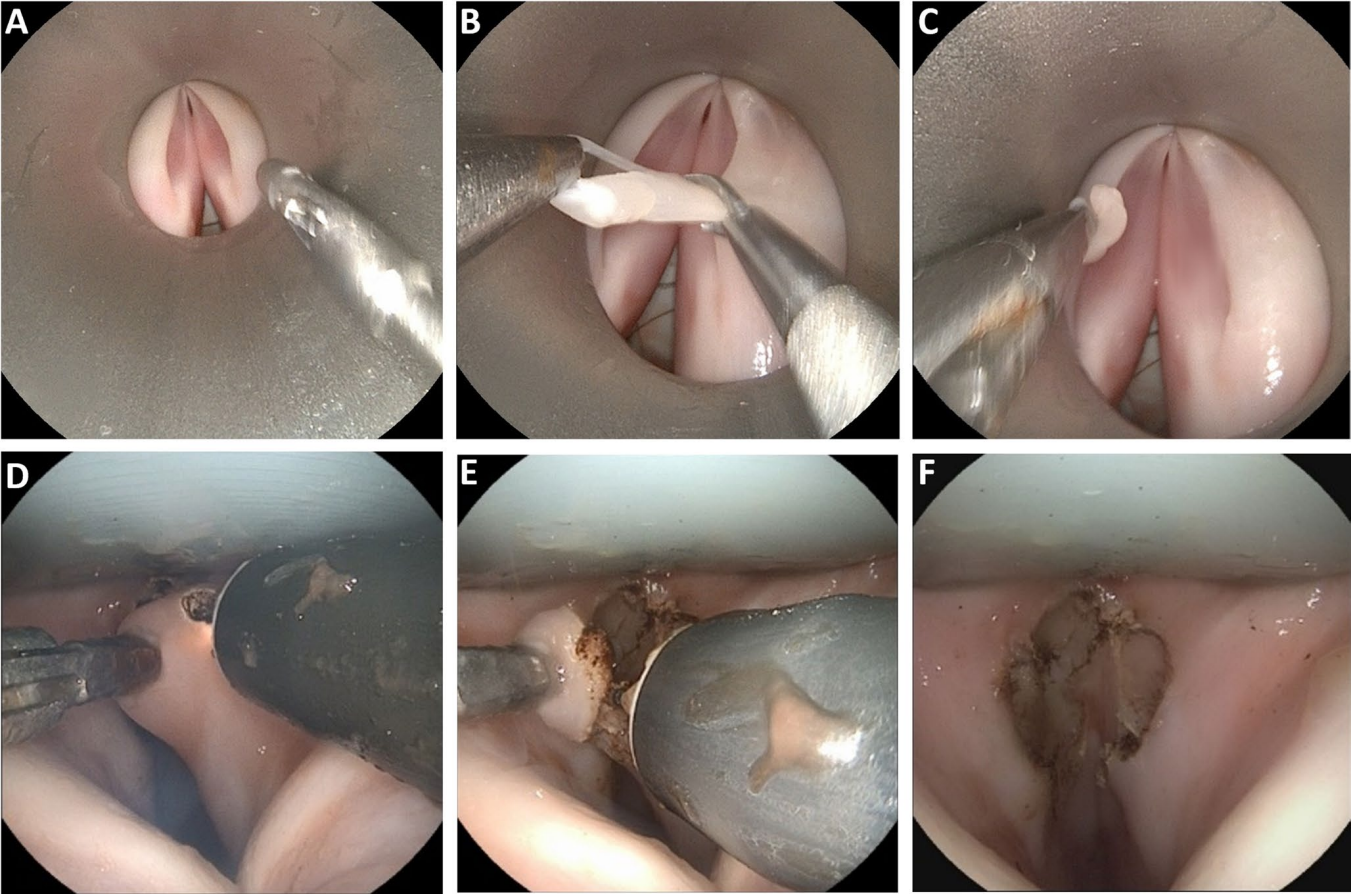
(**A-C**) Performance of a biopsy using a conventional rigid laryngoscope (Karl Storz) and standard microsurgery instruments. (**D-F**) Cordectomy of the right rostral vocal fold using the flexibe grasper instrument and flexible monopolar needle (Lumendi) and the sMAC system. The sMAC system was inserted in the airway manikin with integrated porcine larynx. In this setting the grasper instrument of Lumendi was inserted in the left working channel and the monopolar needle in the right working channel of the sMAC. The pictures were taken by the Storz flexible endoscope which was inserted in the middle channel

Trained surgeons as well as residents were able to perform cordectomies and biopsies using the sMAC system and the conventional operating laryngoscope. To ensure visualization of the surgical site, the 0° endoscope had to be adjusted by an assistant. Figure 5.A-C shows a biopsy of tissue of the right vocal fold performed with the conventional operating laryngoscope. A grasper was used to hold the tissue medially and a scissor with curved blades was used to dissect it (Fig. 5.B). Figure 5 (D-F) depicts a cordectomy of the right vocal fold using the sMAC, therefore, the grasper instrument was inserted into the left working channel of the sMAC to grasp and medialize the right vocal fold (Fig. 5.D). The monopolar needle was inserted to the right working channel. The tip of the monopolar needle is protruded and positioned at the cutting edge (Fig. 5.D). The piece of tissue that has to be resected is pulled out further mediocranially with the grasping forceps and finally dissected with the monopolar needle (Figs. 5.E). The piece of tissue is removed using the grasping forceps. Figure 5.F shows the right vocal cord postoperatively with a corresponding tissue defect. For the left vocal fold, the procedure is reversed accordingly with exchanged instruments.

**Fig. 6:**
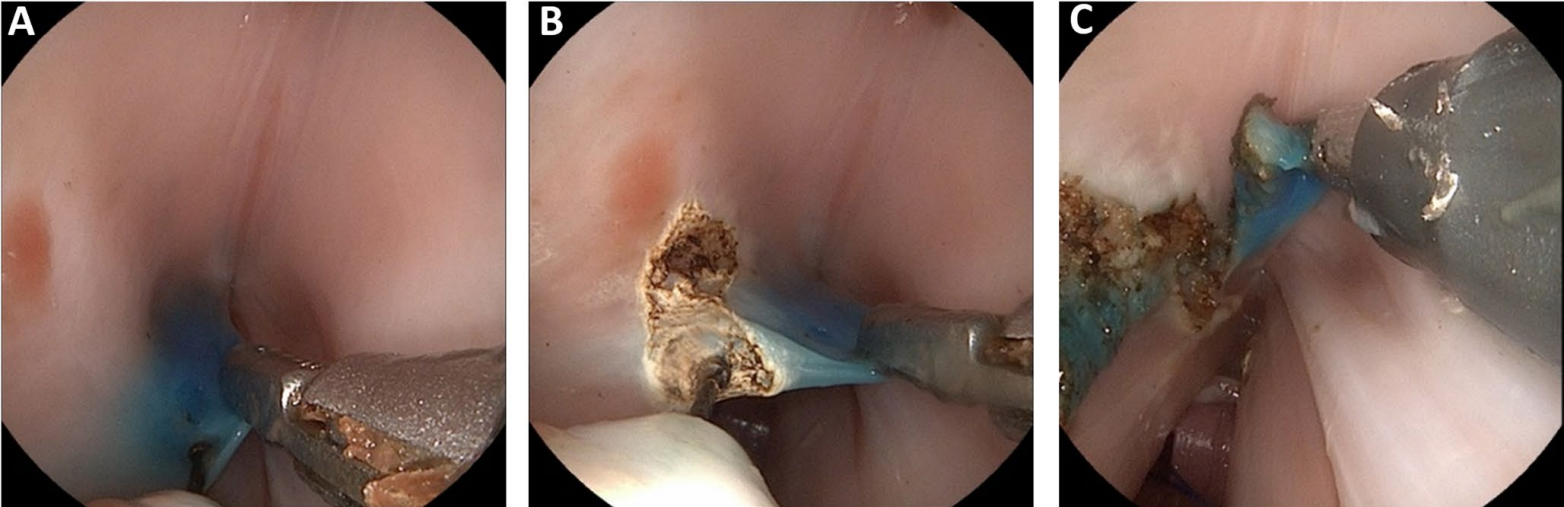
**(A-C)** Shows the resection of blue areas using the sMAC system in three steps. The left vocal fold was prepared by injecting methylene blue with a needle prior to integrating the larynx into the model. The flexible grasper instrument is inserted into the right working channel and pulls the left vocal fold medial. The flexible monopolar needle is inserted to the left working channel and cuts from posterior to anterior

We found that the best resection results with the monopolar needle were achieved with the monopolar mode at a maximal electrical power of 30 watts for forced coagulation and dry cutting. In order to maintain good visualization during coagulation, it is necessary to use a foam aspirator. To simulate a tumor, the vocal folds were prepared by injecting methylene blue with a cannula before integrating the larynx into the model. Then, the blue marked part could be resected as precisely as possible (Fig. 6). To further improve the performance of the operation, the precision of instrument mobility could be improved by reducing the size of the instruments.

The guidance of the residents by experienced tumor surgeons during the operation was very successful in both setups, also due to the good visualization of the surgical field. Intervention by taking over control of the instruments is possible at any time.

## Discussion

This study successfully demonstrates the integration of a porcine larynx into a plastic airway manikin to create a hybrid model for endolaryngeal surgery training.

The model provides a cost-effective, reproducible, and ethically unproblematic alternative that closely approximates clinical conditions. While human cadavers remain the gold standard for anatomical realism, their limited availability, high cost, and regulatory restrictions significantly hinder their routine use in surgical training settings [19-20].

In contrast, porcine larynges are readily available from abattoirs and represent a practical substitute due to their affordability and accessibility. However, users of this hybrid model—both instructors and trainees—must take into account key anatomical differences between porcine and human larynges.

Notably, the porcine larynx features two distinct vocal fold structures: the rostral (vestibular) vocal folds and the caudal (true) vocal folds. The rostral folds are more prominently developed in pigs and, unlike in humans, are capable of oscillation. Histologically, they exhibit a dense composition of collagen and elastic fibers, rendering them more analogous to human true vocal folds. Consequently, the rostral folds are the preferred site for performing surgical interventions such as cordectomies in the training model [17, 20–26]. 

Despite these anatomical distinctions, the overall structure, biomechanical behavior, and tissue handling characteristics of the porcine larynx closely resemble those of the human larynx, supporting its suitability as a surrogate for surgical training in microlaryngeal procedures [19]. 

The approach described here—combining a fixed, pre-fabricated airway trainer with anatomically variable animal specimens—presents certain challenges that must be addressed.

One limitation of the model stems from anatomical variation between specimens (e.g., due to age or gender), which affects the endolaryngeal view and installation process [22]. However, due to the low cost and high availability of porcine larynges, multiple specimens can be prepared in parallel to ensure fitting ones are used.

Careful selection and preparation—including trimming and securing the hyoid and cricoid cartilages—mitigated most inconsistencies.

Over time, significant reductions in preparation time were achieved. From an initial setup duration of approximately 60 min, trained users reduced this to 15–20 min per specimen, particularly when multiple larynges were processed in parallel. This short preparation time makes the model feasible for regular integration into clinical training programs or surgical courses.

Another limitation of the hybrid model relates to the current fixation method. Variability in larynx size can lead to inconsistent fit and stability during installation. While the suture-based caudal fixation and cranial clamping at the hyoid provide basic support, they rely on soft tissue tension and manual adjustment, which may result in inconsistent positioning during surgical manipulation. This can affect both training quality and reproducibility. Repeated clamping may also wear down the manikin. While tape or adhesives might offer temporary stabilization, they risk damaging the plastic over time and reduce repeatability. In the future, improvements such as modular anchoring points or size-adaptive inserts—like 3D-printed adapters or soft silicone liners—could enhance stability, accommodate anatomical variation, and preserve the long-term usability of the model.

Overall, the model setup proved to be highly feasible: tissue fixation was reproducible, exchange of larynges was swift, and overall handling remained efficient throughout the surgical training scenarios.

Both the conventional rigid laryngoscope and the novel hyperangulated surgical MAC (sMAC) system were evaluated using this hybrid model. Residents with limited prior experience in endolaryngeal surgery were able to perform cordectomies and biopsies effectively. Under expert supervision, they demonstrated a steep learning curve facilitated by the model’s consistent anatomical fidelity and realistic tactile feedback. The opportunity for repeated practice without any risk to human patients fostered a safe and supportive learning environment that promotes confidence and skill development.

Feedback from participating residents emphasized the model’s high degree of realism and practical relevance, particularly for surgical novices. The hands-on nature of the training and the authentic tissue response were described as highly beneficial for understanding the spatial and functional anatomy of the larynx. Furthermore, the ability to repeatedly perform the same procedure enhanced procedural fluency and improved instrument coordination.

Simulated pathologies can be introduced by applying visual markers such as colored dyes to the vocal folds, thereby mimicking common clinical findings. This feature enhances the realism of the training session and increases trainee motivation, as the surgical goals can be directly related to real-life operative scenarios. The capacity to replicate target-specific tasks under near-clinical conditions bridges the gap between theoretical knowledge and operative performance.

A limitation of the model lies in its inability to replicate intraoperative complications such as bleeding, tissue fragility, or dynamic changes in patient anatomy. Despite this, the model remains highly effective for teaching core surgical steps and laryngeal anatomy, particularly for residents in the early phases of their surgical training. Overall, the hybrid model supports skill acquisition in a low-risk, high-repetition setting and may help accelerate the transition from theory to clinical practice by enhancing anatomical comprehension, surgical precision, and operative confidence.

To ensure methodological comparability, the hybrid training model was evaluated using both a conventional rigid operating laryngoscope and the novel hyperangulated surgical MAC (sMAC) system. This dual approach not only allowed for direct comparison but also revealed several distinct advantages of the sMAC system—both in terms of its compatibility with the hybrid model and its practical benefits during surgical procedures and training, particularly for less experienced surgeons and in anatomically challenging scenarios.

Designed to conform to the nonlinear geometry of the upper aerodigestive tract, the hyperangulated shape of the sMAC allows transoral access to the glottic plane without requiring the extreme cervical spine extension necessary for rigid laryngoscopy. This feature is of particular relevance in patients with limited neck mobility, trismus, or scarring from prior interventions—clinical situations in which exposure is frequently insufficient when using traditional straight instruments. In contrast to more complex systems such as transoral robotic surgery (TORS), which are limited in their applicability to glottic lesions and often prohibitively expensive, the sMAC provides a low-cost, modular alternative that is scalable, compatible with standard endoscopic infrastructure, and particularly well-suited for resource-limited settings [6, 28–32].

In the hybrid manikin-porcine model, the anatomical challenges could be effectively simulated and addressed. Unlike the rigid laryngoscope, which often required additional adjustment and exerted substantial pressure on the manikin’s oral structures, the sMAC system allowed atraumatic insertion and immediate visualization of relevant laryngeal landmarks. The integrated working channels and flexible endoscopic instrumentation enabled precise bimanual manipulation, even within narrow pharyngeal spaces. This was especially evident in residents with limited experience, who benefited from the intuitive, joystick-based instrument control and the unobstructed view provided by the flexible video endoscope.

From an educational standpoint, the ergonomic advantages of the sMAC system translated into improved focus on surgical tasks, reduced frustration from poor exposure, and enhanced confidence during fine-motor maneuvers. This supports the notion that novel tools such as the sMAC not only expand clinical applicability but also offer significant pedagogical benefits. The use of the hybrid model in conjunction with the sMAC thus presents a compelling scenario for competency-based surgical training: high anatomical realism, low procedural risk, and increased accessibility even in anatomically difficult cases.

While the sMAC system clearly demonstrated substantial educational and ergonomic benefits, it is important to acknowledge that its current design is not without limitations. As with any novel surgical platform, early iterations often reveal technical constraints that must be addressed to fully unlock their clinical and pedagogical potential. The use of the hybrid model in this context provided a valuable testing environment, allowing for the identification of specific shortcomings and opportunities for optimization in both visualization and instrumentation.

Visualization with the 0° optics occasionally required assistance, which means additional personnel during an actual operation or interruption of the progress of the operation if the surgeon has to change and correct the visualization by himself. For this reason, consideration could be given to improving the sMAC with a *chip-on-the-tip* camera solution [27]. Furthermore, while the surgery was possible, smaller instruments might further increase the accuracy for vocal folds procedures. Also, the movability of the monopolar knife could be improved by giving it the same degrees of movement and motion properties as the grasper instrument, which had a joystick for control in all directions on one level. The specially introduced surgical aspirator for smoke removal is somewhat impractical in the clinical practice. Integration into the sMAC via a third working channel or as an external connection option would be desirable when using a monopolar knife.

## Conclusion

In summary, the simulation with this setup is convincingly close to the real situation. Practicing on porcine fresh vocal fold tissue, which is very similar to the human larynx, integrated in a plastic model of a human head, offers, in contrast to a pure plastic model, the possibility to practice surgical procedures and is almost as realistic as a human body donor, while being more readily available and less expensive. The model can be easily reproduced in any department and used for endolaryngeal surgeries as well as for testing different surgical techniques or instruments. However, the clinical utility as training model should be assessed by future studies that i.e., evaluate learning progress for untrained surgeons.

## Data Availability

Data available on request from the authors.
